# Decreasing Heart Rate After Physical Activity Reduces Choking

**DOI:** 10.3389/fpsyg.2020.550682

**Published:** 2020-09-17

**Authors:** Kyoko Hine, Yuto Takano

**Affiliations:** ^1^Department of Computer Science and Engineering, Toyohashi University of Technology, Toyohashi, Japan; ^2^Department of Information Environment, Tokyo Denki University, Tokyo, Japan

**Keywords:** pressure, anxiety, heart rate, physical activity, choking

## Abstract

We occasionally place our bodies under pressure, for example, by playing sports or giving an important presentation at a business meeting. In such situations, most of us have experienced choking, which impairs performance. It has been reported that controlling the heart rate is effective at reducing anxiety, which is one of the causes of choking. Previous studies have proposed a method of reducing choking by undergoing special training for controlling heart rate. Here, we investigated whether a reduction in heart rate after physical activity reduces choking without any special training. Participants bowled under both high-pressure and low-pressure conditions. Before throwing the bowling ball, half of the participants ran on the spot (active condition), whereas the rest of the participants stood instead of running (inactive condition). After controlling for the baseline score, the bowling score in the high-pressure and active condition was significantly better than that in the inactive condition. Additionally, the reduction in heart rate in the active condition was larger than that in the inactive condition. These results suggest that the reduction in heart rate prevented choking without any specific training.

## Introduction

In our lives, we sometimes have to perform on important stages under pressure, such as playing in the finals of a sporting event, playing an instrument in a contest, or giving an important presentation at a business meeting. In such situations, we occasionally experience choking. Choking is a phenomenon in which performance decreases as the importance of the performance is increased (Baumeister, 1984). Finding a way to avoid choking under pressure is desired in many fields. Anxiety induced by pressure is an important factor in evoking choking (Marchant and Gibbs, 2004). In other words, it might be possible to avoid choking by controlling anxiety. In the current study, a method for avoiding choking was investigated by manipulating the cause of anxiety.

It is known that sympathetic nervous system activity increases when a person feels a negative emotion, such as anxiety (Fredrickson et al., 2000). When sympathetic nervous system activity is dominant, heart rate increases (Robinson et al., 1966). On the other hand, parasympathetic nervous system activity increases when a person feels a positive emotion. When parasympathetic nervous system activity is dominant, heart rate decreases. Thus, heart rate is one of the elements affected by feeling an emotion.

Emotions, including anxiety, involve bodily change, such as an increase in heart rate (Friedman and Thayer, 1998). In addition, it was proposed that emotions are accompanied by bodily changes (Damasio, 1999), and humans can identify their own emotional state using their body changes as clues. Strack et al. (1988) demonstrated that participants who held a pen with their mouths reported more humor when they read a cartoon compared to participants who did not hold a pen. This result occurred because the facial expression that one has while holding a pen in their mouth is the same as the facial expression one has while smiling; thus, participants with a pen may have misattributed their facial expression to humor. In other words, humans felt a specific emotion by interpreting a bodily change in a specific situation. Previous studies showed humor (Strack et al., 1988; Chang et al., 2014) as well as other emotional states (Flack et al., 1999; Mori and Mori, 2007) could be felt based on associated bodily changes.

Regarding changes in heart rate, it was also reported that humans felt emotion based on their heart rate. Zillmann et al. (1972) showed that bike riding enhanced aggressiveness compared to sewing because participants may have misattributed the increased heart rate to anger after bike riding. Similar to anger, it might be possible that anxiety is controlled by manipulating heart rate if one misattributes increases or decreases in heart rate to anxiety. Heart rate biofeedback is one of the methods that reduces anxiety training people to decrease their heart rate (Gross, 2002; Hofmann, 2014). In such training, participants practice reducing their heart rate by listening to a coronary tone. A meta-analysis indicated that heart rate biofeedback is effective for reducing anxiety (Goessl et al., 2017).

Although controlling heart rate is effective at reducing anxiety, most of the training requires several practice sessions to decrease heart rate (Sherlin et al., 2009; Ratanasiripong et al., 2012). A situation that evokes choking typically happens suddenly in daily life, and it is likely that individuals under such situations have not taken heart rate biofeedback training. Therefore, it is also necessary to propose a method that does not require specific training and could reduce anxiety. To reduce anxiety without any specific training, we focused on decreasing heart rate after physical activity. It is known that heart rate decreases after peak activity that accompanies increasing heart rate (Cole et al., 1999; Gorelik et al., 2006). If decreasing heart rate reduces anxiety, anxiety might be reduced when one rests after physical activity because the heart rate slows during the recovery period after the activity. In the current study, we investigated whether a reduction in heart rate after physical activity reduced choking. If the reduction of heart rate after activity effectively reduces choking, performance would not be impaired under pressure. To assess the effect of a reduction in heart rate after activity, participants engaged in a game of bowling with high or low pressure, and their performance and heart rate were analyzed.

## Methods

### Participants

Twenty-four participants were recruited for this experiment. The health conditions of the participants were normal based on the participants’ declarations. All participants were randomly assigned to either an active or an inactive condition. Half of them were assigned to the active condition, and the other half were assigned to the inactive condition. Data from four participants were excluded from data analysis because they did not complete the questionnaire or provide heart rate data. Any other criteria regarding inclusion and exclusion of data were applied in the current study. Consequently, data from ten participants (one female and nine males, aged from 20 to 24, mean age = 21.1) for the active condition and another ten participants (four females and six males, aged from 20 to 24, mean age = 21.1) for the inactive condition were analyzed. The *post hoc* analysis using G^∗^Power (Faul et al., 2009) indicated that this sample sizes and experimental design (see below) yielded 80% power to an effect size f of 0.29 (equivalent to partial η2 = 0.08), *p* value of 0.05, correlation among repeated measures of 0.6, and a non-sphericity correction ε of 1. All participants had bowling experience, but no one had formal training or competitive bowling experience. Additionally, none of the participants had received any special training to reduce choking. All participants were informed in writing and signed a letter of consent. This study was approved by the Ethics Committee of Tokyo Denki University. All experiments were conducted in accordance with the Declaration of Helsinki.

### Procedure

All experiments were conducted at a bowling center in Tokyo. Participants were randomly assigned to either the active or inactive condition. All participants in both the active and inactive condition groups completed the high-pressure and low-pressure trials. The order of high/low pressure conditions was counterbalanced across participants; half of the participants first engaged in the high-pressure condition trial and other half participants first engaged in the low-pressure condition trial for each of the active and the inactive conditions to avoid the effect of fatigue or practice. The experiment was conducted individually to avoid an effect of social facilitation (Zajonc, 1965).

**FIGURE 1:**
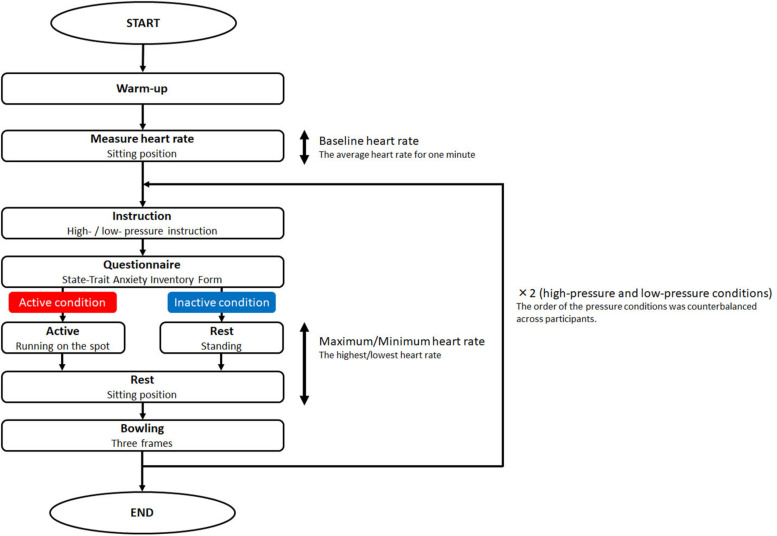
Procedure of the experiment. Participants were assigned to either the active or inactive condition. All participants completed both the high-pressure and the low-pressure conditions. They warmed up and had their baseline heart rate measured. After that, they were given high- or low- pressure instruction, and they then completed the questionnaire. Subsequently, participants in the active condition ran on the spot, whereas participants in the inactive condition were just standing. Then, all participants rested before bowling. After bowling, participants competed under the other high- or low-pressure condition.

Figure 1 shows the procedure of this experiment. At first, participants practised bowling for two frames as a warm-up. After getting enough rest, participants wore a heart rate sensor (POLAR, H10) around their chest, which was coupled to an iPod touch (Apple). This sensor recorded heart rate each second. The participants’ heart rates at this point were considered the baseline. The baseline data were collected over a 1-min period of relaxation in a sitting position. Participants kept wearing the sensor until the end of the experiment.

In the high-pressure condition, participants were given instructions about the video recorder, the competition, performance-contingent rewards, and evaluation by a professional player. A previous study reported that this information successfully manipulated pressure (Wang et al., 2004). In the low-pressure condition, these instructions were not given. After giving the instructions, they answered questions from the State-Trait Anxiety Inventory Form Y-1 in Japanese (Hidano et al., 2000) to assess their state anxiety (Spielberger, 2010). After completing the questionnaire, participants in the active condition ran on the spot for 40 s. Participants in the inactive condition stood instead of running. Then, they sat down for 20 s. After resting, they bowled for three frames. They were told that they had to do their best to knock down as many pins as they could. In an exploratory experiment, we checked the stability of the score in one game. Over three frames, the score became unstable because of participant fatigue. Therefore, we decided that three frames of bowling as one game was suitable for the current experiment. After the three frames of bowling, participants then completed the other high- or low-pressure condition. The experiment took 1 h. Participants were finally thanked and debriefed.

## Results

### Scores on the State-Trait Anxiety Inventory Form Y-1

To examine state anxiety, the scores on the State-Trait Anxiety Inventory Form Y-1 in Japanese (Hidano et al., 2000) were calculated. The average of the scores for the high-pressure condition (45.6, *SD* = 7.9) was significantly higher than that for the low-pressure condition (36.6, *SD* = 6.3) [*t*(18) = 2.60, *p* = 0.02, *r* = 0.52]. Therefore, anxiety was successfully manipulated in the current study. In addition, there was no significant difference in the score between the active (high-pressure: average = 43.2, *SD* = 6.3; low-pressure: average = 37.0, *SD* = 5.6) and inactive condition (high-pressure: average = 44.9, *SD* = 9.7; low-pressure: average = 36.0, *SD* = 7.2) for the high-pressure [*t*(18) = 0.46, *p* = 0.65, *r* = 0.11] and the low-pressure condition [*t*(18) = 0.34, *p* = 0.74, *r* = 0.08]. Additionally, there was no significant difference between females (high-pressure: average = 47.6, *SD* = 4.3; low-pressure: average = 39.0, *SD* = 9.2) and males (high-pressure: average = 42.9, *SD* = 8.9; low-pressure: average = 35.5, *SD* = 5.7) for the high-pressure [*t*(18) = 1.16, *p* = 0.26, *r* = 0.26] and the low-pressure condition [*t*(18) = 0.95, *p* = 0.34, *r* = 0.22]. The results indicated that the participants did not differ in anxiety across the active/inactive conditions or across sex.

### Bowling Score

First, the bowling scores, which were the number of pins knocked down, were calculated for each condition. In this experiment, the bowling task consisted of three frames. Therefore, the maximum bowling score was 50. To check the performance at the baseline in the active and the inactive condition, the bowling scores in the low-pressure condition were compared in the active and inactive conditions. The score in the low-pressure condition was regarded as the baseline because the low-pressure condition was similar to that in an ordinary bowling situation. The score in the active condition (average = 26.1, *SD* = 7.6) was not significantly different from that of the inactive condition (average = 27.8, *SD* = 7.3) [*t*(18) = 0.66, *p* = 0.45, *r* = 0.15]. Therefore, the performance in the baseline was not different in the active and inactive conditions.

**FIGURE 2:**
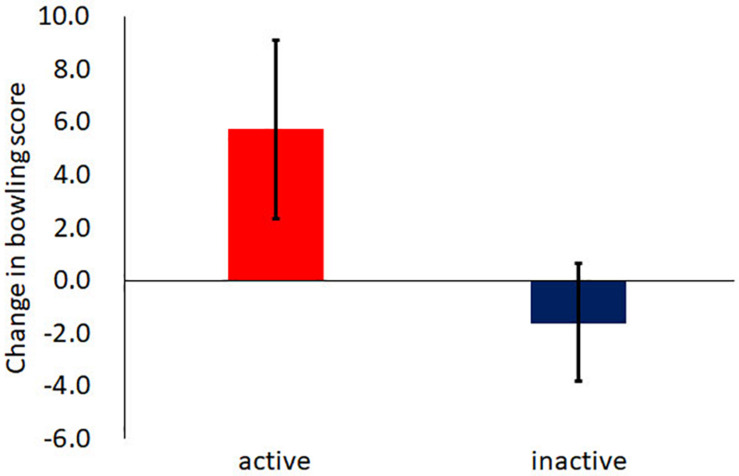
The average change in bowling scores. The change was calculated as the performance in the low-pressure condition subtracted from the high-pressure condition. The error bar indicates the standard error.

Next, the change in the bowling score was calculated as the bowling score in the low-pressure condition subtracted from the score in the high-pressure condition. The change in the bowling score indicated how much pressure changed the bowling score. A positive value indicated that pressure enhanced the bowling score whereas a negative value indicated that pressure inhibited the score. Figure 2 shows the average changes in bowling scores for each condition. The average change for the active condition (5.7, *SD* = 7.1) was significantly higher than that for the inactive condition (−1.6, *SD* = 10.7) [*t*(18) = 2.08, *p* = 0.05, *r* = 0.44].

### Heart Rate

The baseline heart rate was calculated as the average heart rate during 1 min after the warm-up session. The baseline heart rate in the active condition (average = 95.7, *SD* = 13.9) was not different from that in the inactive condition (average = 91.2, *SD* = 11.5) [*t*(18) = 0.71, *p* = 0.49, *r* = 0.17]. Therefore, the baseline heart rate was the same in the two conditions.

**FIGURE 3:**
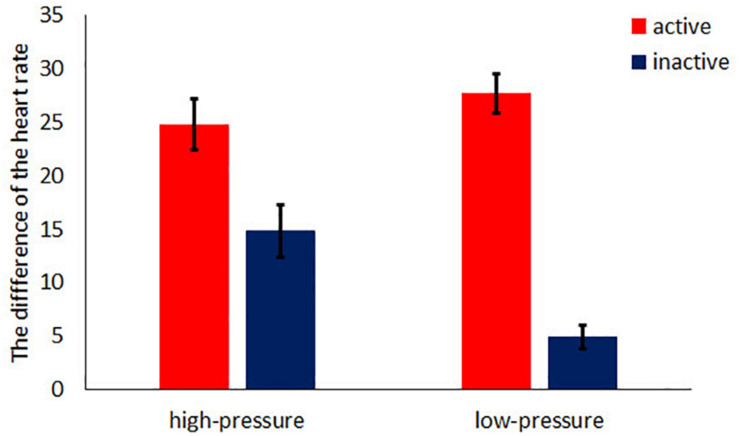
The average difference in heart rate (the maximum heart rate – the minimum heart rate). The error bar indicates the standard error.

To assess the effect of the reduction in heart rate, the differences in heart rate were calculated as the minimum heart rate subtracted from the maximum heart rate observed for 1 min (running/standing for 40 s and sitting for 20 s) before participants went bowling. All maximum heart rates were observed during running/standing for 40 s, and all minimum heart rates were observed during sitting for 20 s. Figure 3 shows the average differences in heart rate for each condition. Two-way analysis of variance (ANOVA) was conducted on the difference in heart rate using activity (active or inactive) as the between factor and pressure (high-pressure or low-pressure) as the within factor. A main effect of activity was found [*F*(1,18) = 47.26, *MSE* = 2689.60, *p* = 0.00, partial η^2^ = 0.53]. A main effect of pressure was not found [*F*(1,18) = 2.74, *MSE* = 122.5, *p* = 0.11, partial η^2^ = 0.03]. A significant interaction was found [*F*(1,18) = 9.17, *MSE* = 409.6, *p* = 0.01, partial η^2^ = 0.08]. For both high-pressure and low-pressure conditions, the main effect of activity was found [high-pressure condition: *F*(1,18) = 9.85, *MSE* = 500.0, *p* = 0.00, partial η^2^ = 0.23; low-pressure condition: *F*(1,18) = 51.18, *MSE* = 2599.2, *p* = 0.00, partial η^2^ = 0.68]. This result indicates that the reduction of heart rate in the active condition was larger than that in the inactive condition for both high- and low- pressure conditions.

**FIGURE 4:**
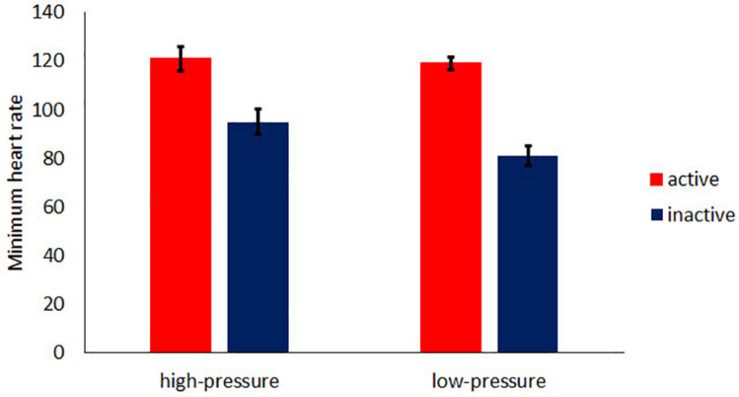
The average of minimum heart rate. The error bar indicates the standard error.

To investigate the effect of the minimum heart rate that was recorded just before the bowling game, ANOVA was conducted on the minimum heart rate using activity (active or inactive) as the between factor and pressure (high-pressure or low-pressure) as the within factor. Figure 4 shows the average of minimum heart rate for each condition. A significant interaction was found [*F*(1,18) = 4.04, *MSE* = 360.00, *p* = 0.05, partial η^2^ = 0.02]. For the inactive condition, a main effect of pressure was found [*F*(1,18) = 10.84, *MSE* = 966.10, *p* = 0.00, partial η^2^ = 0.33], whereas a main effect of pressure was not found in the active condition [*F*(1,18) = 0.00, *MSE* = 18.10, *p* = 0.66, partial η^2^ = 0.01]. Additionally, in both high-pressure and low-pressure conditions, the minimum heart rate in the active condition was significantly higher than that in the inactive condition [high-pressure: *F*(1,18) = 15.66, *MSE* = 3354.10, *p* = 0.00, partial η^2^ = 0.63, low-pressure: *F*(1,18) = 33.50, *MSE* = 7182.10, *p* = 0.00, partial η^2^ = 0.52].

## Discussion

This study investigated whether a reduction in heart rate after physical activity reduced choking. The obtained results demonstrate two significant findings. First, the reduction in heart rate after physical activity reduced choking. The change in the bowling score in the active condition was higher than that in the inactive condition. Additionally, the difference in heart rate in the active condition was larger than that in the inactive condition. These results suggest that a rest-induced reduction in heart rate after physical activity could prevent choking. One might think that performance rather than choking was affected by the activity before bowling. However, the performance at baseline (in the low-pressure condition) in the active condition was not significantly higher than that in the inactive condition. Therefore, the possibility that the activity before bowling boosted performance itself could not fully account for the current results, and there is still the possibility that reduction of heart rate after the activity reduces choking. Methods for avoiding choking have been proposed previously. For example, “quiet eye” (Vickers, 1996) is one of the well-known ways of avoiding choking, in which a player’s attention is controlled. Most studies have reported that the quiet eye affects player performance with special training (Vine et al., 2011; Causer et al., 2014). Taking a rest after a physical activity is a method in which a player’s heart rate is controlled without any special training and reduces their anxiety consciously.

Second, the reduction in heart rate in the active condition was larger than that in the inactive condition. On the other hand, the minimum heart rate in the active condition was higher than that in the inactive condition. These results indicate that the reduction in heart rate rather than a low heart rate is an important factor in avoiding choking caused by anxiety. Appraisal theory (Seth, 2013), in which emotion emerges from cognitive evaluations of physiological changes. In the current study, participants in the active condition might have evaluated their reduction in heart rate as a reduction in their anxiety even if the actual cause of the altered heart rate was taking a rest after physical activity. Further studies are required to clarify how emerging emotions are evaluated. One might think that the activity disrupted the cognitive evaluation, and the faulty evaluation led to the reduction in anxiety. If so, in the low-pressure condition, the performance in the active condition should differ from that in the inactive condition because the activity disrupted the evaluation of their anxiety. However, as mentioned above, the performance in the low-pressure condition in the active condition was not different from that in the inactive condition. Therefore, the disruption by the activity could not fully account for the results in the current study, and there is still the possibility that the reduction in heart rate moderated anxiety.

In this study, the change in bowling score was positive for the active condition, which meant that the high-pressure and active condition boosted bowling performance. On the other hand, for the inactive condition, the change in bowling score were negative, which indicated that the high-pressure and inactive condition disrupted bowling performance. Pressure sometimes improves performance (Otten, 2009), and sometimes it does not. What determines whether pressure leads to success or failure? It was reported that there was a negative correlation between performance and self-focus in which participants focusing internally showed disrupted performance (Liao and Masters, 2002). In the current study, reductions in heart rate might make participants focus away from themselves. Another study proposed that perceived control, that is, knowledge derived from accurate predictions of subsequent stimuli (Seger, 1994), improved performance (Otten, 2009). Participants in the active condition in this study might have assigned their cognitive resources to perceived control instead of self-focus. Further studies are required to clarify the factors that enhance/disrupt performance under pressure.

This study revealed that the reduction in heart rate after physical activity affects a performance under high pressure. However, there are still limitations to explore the effect of the reduction in heart rate on the performance. For the inactive condition, the reduction of heart rate in the high-pressure condition was larger than that in the low-pressure condition. If the large reduction in heart rate induced the higher performance on the bowling score, the change in bowling score, which was calculated as the performance in the low-pressure condition subtracted from the high-pressure condition, should be positive value. However, such a result was not obtained in this study. Therefore, it is still unclear which affected on the performance under high pressure, the reduction in heart rate after physical activity (quality of reduction in heart rate) or difference of decrement of heart rate between the active and inactive conditions (quantity of reduction in heart rate). In further studies, it is required to clarify how reduction in heart rate after physical activity affect to avoid choking.

Another limitation was related to participant’s attributes. In the current study, all participants were young people (aged from 20 to 24), and the number of male participants was larger than that of female participants (Fisher’s exact test; *p* = 0.04). It was reported that age and gender affect the average heart rate (Sagie et al., 1992; Singh, 1999; Mason et al., 2007). In further studies, getting data from various attribution participants could lead the results of the current study more reliable.

These results suggested that reducing heart rate prevented choking without any specific training. Increasing heart rate by physical activity and then resting seems to be a relatively easy way to prevent choking. This study contributes not only to sports science but also to daily tasks performed under high pressure.

## Data Availability Statement

The raw data supporting the conclusions of this article will be made available by the authors, without undue reservation.

## Ethics Statement

The studies involving human participants were reviewed and approved by the Ethics Committee of Tokyo Denki University. The patients/participants provided their written informed consent to participate in this study.

## Author Contributions

KH designed the study and wrote the manuscript. YT designed the research and collected and analyzed behavioral data. Both authors discussed the results and commented on the manuscript.

## Conflict of Interest

The authors declare that the research was conducted in the absence of any commercial or financial relationships that could be construed as a potential conflict of interest.
